# Mechanisms of cellular fibrosis associated with cancer regimen-related toxicities

**DOI:** 10.3389/fphar.2014.00051

**Published:** 2014-03-27

**Authors:** Maria L. Mancini, Stephen T. Sonis

**Affiliations:** Biomodels LLC, WatertownMA, USA

**Keywords:** fibrosis, radiation therapy, chemotherapy toxicity, oral mucositis, radiation dermatitis, proctitis, pulmonary fibrosis, TGF-β

## Abstract

Fibrosis is a common, persistent and potentially debilitating complication of chemotherapy and radiation regimens used for the treatment of cancer. The molecular mechanisms underlying fibrosis have been well studied and reveal overall processes that are largely ubiquitous. However, it is important to note that although the processes are similar, they result in cellular phenotypes that are highly tissue specific. These tissue specific differences may present opportunities for therapeutic interventions to prevent or treat this often irreversible condition. Data generated from animal models of cancer therapy-related tissue toxicities have revealed that the signaling pathways involved in fibrosis are the same as those involved in the normal injury response and include the transforming growth factor β superfamily and a range of pro-inflammatory cytokines. The critical difference between normal wound healing and fibrosis development appears to be, that in fibrosis, these signaling pathways escape normal cellular regulation. As a result, an injury state is maintained and processes involved in normal healing are usurped. There are a few, if any, therapeutics that effectively prevent or treat fibrosis in patients. Consequently, cancer survivors may be chronically plagued with a variety of life-altering fibrosis-related symptoms. Uncovering the signaling pathways that drive cellular fibrosis is paramount to the development of specific therapeutics that will mitigate this potentially devastating condition.

## INTRODUCTION

Radiation and chemotherapy remain the most commonly used therapies for the treatment of multiple types of human cancer. While these therapies have been met with great success in the treatment of tumors, they are known to induce a wide range of acute and chronic toxicities. These regimen-related toxicities are not only associated with poor health outcomes, but they often become dose-limiting for patients and impair patients’ quality of life (QoL) and recovery in both the short and the long term (Elting et al., 2008). Hematological disorders such as anemia, thrombocytopenia, and neutropenia are among the most common complications associated with radiation and chemotherapy; however, cancer patients are also at risk for a wide range of non-hematological toxicities (Sonis and Keefe, 2013). The overall incidence of some form of cancer treatment-related toxicity is almost 100% and can occur both during cancer treatment (acute toxicities), or will develop well after the completion of treatment (≥100 days, late toxicities). Understanding chemotherapy and radiation induced toxicities is of high importance due to their direct impact on patients’ symptoms and QoL and their high resource and financial burden. This review will focus on regimen-related toxicities that are associated with cellular fibrosis of epithelial tissues and include radiation and/or chemotherapy-induced fibrosis of the gastrointestinal tract (oral mucositis and proctitis), the skin (dermatitis), and the lung (pulmonary fibrosis).

## MECHANISMS OF FIBROSIS

Within the past decade, there has been a major shift in the conventional paradigms associated with the pathogenesis of regimen-related toxicities in cancer patients. Historically, normal tissue damage was attributed to the concept that since radiation or chemotherapy could not distinguish between rapidly dividing cancer cells and rapidly dividing normal cells the result was non-specific clonogenic cell death. Not only were the cellular kinetics associated with normal tissue toxicity inconsistent with this hypothesis, but it failed to address damaging changes to peripheral tissue such as subepithelial connective tissue or muscle (i.e., heart) and completely ignored non-tissue based complications like fatigue, cognitive dysfunction or cachexia. Accumulating evidence suggests that, while some clonogenic cell death does occur, the bulk of pathogenesis is the consequence of a sequence of related biological events that result in both direct and indirect tissue and systemic damage mediated by a diverse range of canonical pathways. Of particular interest has been the finding that the temporal genomic characterization of these toxicities has shown their compatibility with conditions having similar phenotypes including chemotherapy-induced diarrhea and inflammatory bowel disease (IBD), chemotherapy-fatigue and chronic fatigue syndrome, regimen-related cognitive dysfunction, and Alzheimer’s disease. While this observation has not been surprising, it does serve to emphasize the potential impact of better understanding fibrosis in the context of cancer treatment, as well as possibly opening treatment intervention opportunities beyond the oncology population.

**FIGURE 1:**
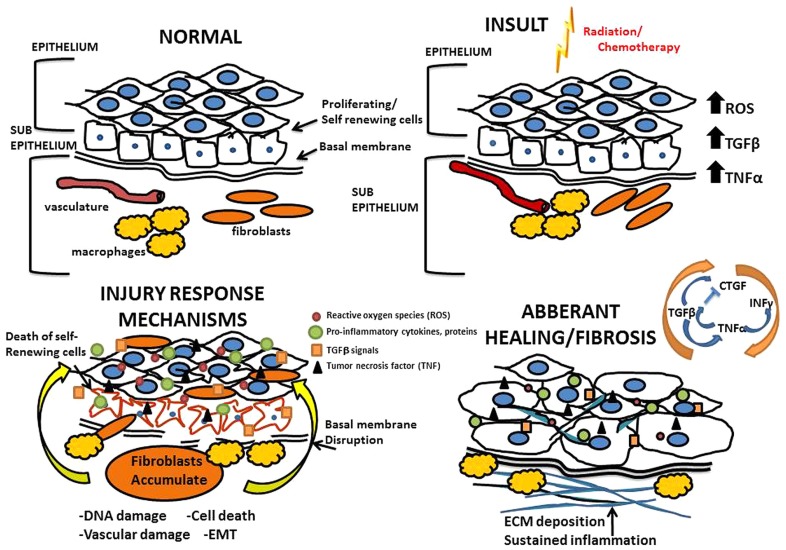
**Mechanisms of radiation and chemotherapy-induced fibrosis.** Above is an illustration of the processes that drive fibrosis detailing the mechanisms governing the overall tissue changes that occur as a result of radiation and chemotherapy induced injury; progressing from normal tissue to an eventual fibrotic state.

Data outlining molecular mechanisms by which treatment-related fibrosis develops has largely been captured from animal models that accurately replicate chemotherapy or radiotherapy regimens routinely used in patients (Sonis and Keefe, 2013). Immediately following insult due to chemotherapy and radiation, there is a large cellular response that involves cell type specific programs occurring in three general phases (**Figure 1**). First is the inflammatory phase, where inflammatory cells are recruited and release cytokines to recruit fibroblasts and other immune cells to the site of injury. Second is the proliferative phase, which is characterized by fibroblasts proliferating and migrating to the site of injury where they form a scaffold on a temporary fibronectin matrix present in the tissue and deposit collagen type III to form a new barrier. Third is the remodeling phase which lasts several weeks and involves building up the new extracellular matrix (ECM) by the converting the flexible collagen type III into the more permanent collagen type I. This conversion is mediated through both secreted proteases and matrix building proteins from local fibroblasts. Not surprisingly, heterogeneity exists among the various fibroblast populations recruited; fibroblast subsets have specialized functions and vary in rates of proliferation, response to inflammatory signals and ECM production (Sempowski et al., 1995).

Injury resulting from radiation and chemotherapy is initiated through two major paths: radiolytic hydrolysis and stimulation of the innate immune response. Of the two, oxidative stress is the best studied with respect to cancer treatment-associated tissue injury. Radiation or chemotherapy-induced oxidative stress leads to the production of oxygen free-radicals; specifically the reactive oxygen species (ROS) superoxides, hydrogen peroxides, and hydroxyl radicals that cause oxidative damage to the tissue (Pan et al., 2012). Once damage to the tissue has been initiated, inflammatory cells are recruited to the injured area, a process orchestrated by vasodilation and vascular permeability. On the cellular level, fibrosis involves the coordination of a variety of cell types largely mediated through the fibroblast. The infiltrating immune cells secrete cytokines that drive the differentiation of fibroblasts and other self-renewing cells into myofibroblasts which deposit collagens and other ECM proteins at and around the site of tissue damage (Eckes et al., 2000; Wang et al., 2011). Under normal circumstances of wound repair, the expanded ECM would be provisional until the process of re-epithelialization occurs. When the wound repair process is deregulated, and re-epithelialization is prevented, fibrosis occurs (Eckes et al., 2000; Ueha et al., 2012). Until recently it was generally accepted that fibrosis occurs in a confined space only affecting the area immediately surrounding the site of injury. It has since become clear that fibrosis arises and persists systemically. Circulating immune cells, chemokines, and bone marrow derived fibroblasts are recruited to sites of injury generating and depositing excess ECM proteins (Andersson-Sjöland et al., 2011). On a molecular level, the processes of fibrosis are driven by transforming growth factor beta (TGFβ), connective tissue growth factor (CTGF), tumor necrosis factor alpha (TNF-α), and interferon gamma INF-γ (Reviewed in Leask and Abraham, 2004). Susceptible organs include any connective tissue containing organ and/or tissues containing mesenchymal cells that are capable of differentiating into fibroblasts. While the initial insult is often focused on a specific organ/tissue, systemic effects are seen in distant sites highlighting the widespread nature of regimen-related toxicities. For example patients receiving radiation therapy (RT) with or without cytotoxics for head and neck cancer develop diarrhea, supporting the idea that even directed therapies are not contained to the treated tissues (Vermorken and Specenier, 2010).

The TGFβ signaling axis drives the majority of the cellular events associated with radiation-induced fibrosis (Boerma et al., 2013). The TGFβ superfamily regulates a wide variety of cellular processes in response to injury including survival, proliferation, and migration (Reviewed in Massague, 2012). TGFβ binds to its cognate receptors and channels its instructive signals through the Smad family of transcription factors inducing the expression of target genes involved in the cellular phenotypes described above and also upregulates the genes responsible for collagen synthesis (Ghosh et al., 2008). In addition, signaling through this pathway drives cellular dedifferentiation and reprogramming. Multiple studies demonstrate that epithelial cells can undergo epithelial to mesenchymal transition (EMT), acquire the characteristics of fibroblasts and subsequently differentiate into myofibroblasts. This transition is driven by transcriptional changes that are mediated by TGFβ signaling and ultimately further exacerbate tissue fibrosis (Venkov et al., 2007; Wang et al., 2011). Taken together, TGFβ acts both as a potent chemoattractant for fibroblasts and subsequently a mediator of proliferation, migration, differentiation, and ECM deposition of the recruited fibroblasts at the site of injury.

The molecular mechanisms of TGFβ signaling associated with the development of fibrosis have been underscored through genetically modified animal models (Chen et al., 2001; Flanders et al., 2002; Chan et al., 2012; Balli et al., 2013). Epithelium in animals engineered to suppress Smad3 has a reduced fibrotic response (Flanders et al., 2002; Oh et al., 2012). Furthermore, Smad3 null animals demonstrate accelerated wound repair characterized by enhanced re-epithelialization with a reduced inflammatory response (Ashcroft et al., 1999). While the data from these studies suggest that TGFβ/Smad signaling is directly involved in the induction of fibrosis, other studies have shown that signaling through this pathway is not required for maintaining fibrosis and therefore therapies directed at regulating Smad activity would likely have to be administered as an early intervention (Holmes et al., 2001; Leask et al., 2003). To further support this notion, the findings of a recent meta-analysis of clinical data failed to demonstrate an association between three SNPs commonly associated with TGFβ1 and the risk of late radiation-induced normal tissue damage (Zhu et al., 2013).

CTGF is induced by TGFβ, specifically via Smad enhancer elements in the CTGF promoter, and therefore acts as a downstream effector of TGFβ mediated fibrosis (Leask et al., 2003). Data suggest that after initial induction, expression of CTGF remains constitutive in areas of fibrosis and escapes regulation by TGFβ signaling (Holmes et al., 2001). Therefore cellular events downstream of CTGF secretion, including induction of collagen I expression in neighboring cells, may contribute to the maintenance of fibrosis. Furthermore, the paracrine nature of CTGF activity suggests a pivotal role in inducing fibrosis in surrounding tissues further exacerbating the duration and severity of fibrotic phenotypes (Sonnylal et al., 2013). One mechanism of negative regulation of these signaling events involves the pro-inflammatory cytokine TNF-α which acts as an inhibitor of TGFβ induced CTGF expression and therefore has anti-fibrotic activity (Mori et al., 2002). In addition, endogenous TNF-α applied directly to injured skin reduced the deposition of collagen. Some data suggests however, in certain situations sustained TNF-α expression may contribute to fibrosis in alternative mechanisms, through directly stimulating the proliferation of fibroblasts (Piguet et al., 1990; Leask and Abraham, 2004). That TNF-α does not regulate CTGF expression directly further supports the importance of CTGF related signaling events downstream of TGFβ in propagating fibrosis.

Increased expression of the pro-inflammatory cytokine INF-γ goes hand in hand with tissue fibrosis. Naïve CD4+ T helper (Th) cells differentiate into Th1 and Th2 subsets that secrete cytokines in response to inflammation including INF-γ and a plethora of interleukins essential for immune cell functions (Chen et al., 2001). The specific role of INF-γ is incompletely understood; it is unclear whether the presence of INF-γ at sites of fibrosis confers a role that is promoting or reparative. One study demonstrated that animals deficient in INF-γ did not develop pulmonary fibrosis resulting from treatment with Bleomycin suggesting a critical role for this cytokine in promoting fibrosis (Chen et al., 2001). By contrast, other studies have shown that INF-γ has anti-fibrotic activity mediated through suppression of collagen synthesis by fibroblasts and inhibition of TGFβ expression (Gurujeyalakshmi and Giri, 1995). Therefore, data suggest that spatiotemporal expression of INF-γ may determine the role it plays in the development and maintenance of fibrosis. This is similar to the dichotomous role for TNF-α, the pro-inflammatory cytokine that is also regulated by and synergizes with INF-γ (Nathan et al., 1984). Taken together the signaling events regulating fibrosis represent distinct yet interdependent signaling pathways that become de-regulated in situations of radiation and chemotherapy-induced injury. These pathways provide options for therapeutic interventions (**Table 1**) and will be categorically explained in greater detail.

**Table 1:** Current standard of care therapies for common regimen-related toxicities.

Regimen-related toxicity	Treatment	Mechanism
Oral mucositis	MuGuard, gelclair	Promotes barrier function
	Caphosol, kepivance	Topical biologics designed to promote healing		
Proctitis	5-ASA, steroids	Anti-inflammatory
	Sucralfate, metronidazole	Promotes barrier function, prevents bacterial colonization		
Dermatitis	Aloe vera	Largely unknown; limited barrier function and lubrication		
Pulmonary fibrosis	Amifostine	Produces cytoprotective thiols
	Superoxide dismutases (SODs)	Dismutate superoxides; prevents oxidative damage

*Examples of therapies used to treat patients suffering with the toxicities described in this article. None of these therapies are specifically targeted to address fibrosis directly; rather they are designed to prevent the initial inflammatory responses that eventually lead to fibrosis for purposes of prevention. This strategy has been met with limited success and therefore there is an unmet clinical need for development of therapeutics focused specifically on fibrosis.
*

## FIBROSIS ASSOCIATED WITH ORAL MUCOSITIS

Radiotherapy is a treatment mainstay for cancers of the head and neck and is typically administered concomitantly with radiosensitizing doses of chemotherapy, most commonly Cisplatin (Sonis, 2011). The oral, oropharyngeal, hypopharyngeal, and laryngeal mucosa are often included in the radiation field. As a consequence severe tissue injury in the form of mucositis is virtually ubiquitous. Lesions of oral mucositis consist of diffuse, deep, extremely painful ulcers involving the mouth’s movable mucosa (Sonis, 2013). As in most treatment paradigms, radiation is in small, daily fractionated doses of 2Gy, 5 days per week, for cumulative doses of 60–70Gy (Silverman, 2007). Chemotherapy is given either every 3 weeks during the radiation period (days 0, 21, and 42) or weekly, but in smaller doses. These treatment schemes induce a predictable pattern of mucositis. By the end of the first week of treatment (cumulative radiation dose of 10Gy) erythematous changes start to occur and are accompanied by a level of pain described as being comparable to a bad food burn. By cumulative doses of 30Gy, ulceration develops (Silverman, 2007; Sonis, 2013). Unlike the typical mouth sores of aphthous stomatitis (canker sores), ulcerative lesions associated with mucositis are more broad and deep. Consequently, they are disproportionately painful requiring opioid analgesics which are often ineffective. Patients frequently are unable to eat by mouth and require gastrostomy tube placement for feeding (Sonis, 2007). Subepithelial changes that accompany mucositis predispose patients to fibrosis and the clinical development of trismus, which is characterized by a restricted ability to open the mouth sometimes referred to as “lock jaw” (Lyons et al., 2013). Of regimen-related tissue injuries, the pathogenesis of mucositis is probably best understood and has been described as a 5-phase algorithm which it appears to share with other types of cancer regimen-induced epithelial damage (Sonis et al., 2000). While originally directed at the oral mucosa, it is now clear that the biological sequence is the same for regimen-related damage throughout the gastrointestinal tract.

As noted above, the initiation phase is characterized by oxidative stress and activation of the innate immune response which largely occurs in the endothelium and connective tissue of the submucosa after exposure to radiation (Sonis, 2007). In response to the activation of a range of canonical pathways, a cytokine cascade follows as does simultaneous fibronectin breakdown, and amplification of pro-inflammatory cytokine signaling cascades in the aforementioned TGFβ and TNF-α pathways. Ultimately the epithelium breakdowns down and ulceration occurs (Sonis et al., 2000). Secondary bacterial colonization and breaks in the mucosal barrier allow for penetration of whole bacteria or, more commonly, cell wall products which activate infiltrating macrophages to produce additional cytokines. The injured mucosa undergoes extensive remodeling to seal off the tissue to try and prevent invasion of the bacteria. This process involves cellular deposits of ECM that, as with many situations of wound repair, can encompass the surrounding tissue and result in extensive fibrosis.

Clinically, patients with fibrosis of the mouth develop trismus and cannot function normally (Lyons et al., 2013). But fibrosis is not limited to the mouth. Salivary function is obscured by replacement of parenchyma with fibrous tissue and esophageal strictures can develop (Fujita-Yoshigaki and Qi, 2009). While mucositis is an acute toxicity, fibrosis-related changes tend to be more chronic and are thus increasingly significant as cancer survivorship improves. Animal models of mucositis in rats, mice and hamsters have provided highly useful templates to define the pathogenesis of mucosal injury and evaluate the potential efficacy of new interventions. Among the models routinely used, a highly translatable model in hamsters has been especially useful in drug development and in demonstrating the relationship between radiation-induced mucosal damage and the development of fibrosis (Ara et al., 2008; Murray et al., 2010). An approved, effective mechanistically based therapy for radiation-induced mucositis is being aggressively sought. It is likely that a halo benefit from such a treatment will be attenuation of fibrosis development.

## FIBROSIS ASSOCIATED WITH PROCTITIS

Radiation-induced proctitis is a complication resulting from radiation directed at the lower abdomen or pelvis typically associated with rectal, prostate, or cervical malignancies. The incidence of proctitis is approximately 75% with symptoms consisting of rectal bleeding, mucus production and diarrhea (Counter et al., 1999). Not surprisingly given the nature of rectal epithelium, symptoms begin within 2–3 weeks after the start of treatment. In contrast to lesions of the upper gastrointestinal tract, however, proctitis often lasts anywhere from a few months to several years following the completion of radiation therapy. The stages are multifactorial beginning with initial damage to the mucosa, followed by delayed slow growing connective tissue and finally a tissue response to vascular ischemia (Okunieff et al., 2005). In addition to rectal bleeding and mucus, patients suffering from proctitis also experience tenesmus, or a feeling or inability to empty the bowel upon defecation. This occurs as a result of epithelial fibrosis in the rectum due to radiation exposure, a condition which is often permanent and irreversible (Symon et al., 2010).

The overall pathogenesis of fibrosis associated with radiation-induced proctitis is similar to that which occurs in oral mucositis. However, unlike oral mucositis, radiation-induced proctitis has not been as aggressively studied pre-clinically and there exists a very high unmet clinical need in this area. Proctitis is initiated on the cellular level with apoptosis, disruptions of mitosis, and fibroblastic proliferation that leads to swelling and sloughing of the rectal mucosa (Haboubi et al., 1988; Brunn and Fletcher, 2006). It has become increasingly clear that the complications associated with proctitis involve coordination between the processes of fibrosis and angiogenesis. The cellular alterations to the vasculature including neovascularization and telangiectasias lead to clinical symptoms of persistent bleeding. Increased fibrosis causes ischemia and eventual necrosis of the bowel tissue (Haselton et al., 1985; Fajardo, 2005). The exact mechanisms for the late changes in vascularity and fibrosis have yet to be elucidated, however, there is evidence that several growth factors including platelet-derived growth factor, vascular endothelial growth factor, and fibroblast growth factor play key roles in the pathology (Brunn and Fletcher, 2006) More recent evidence has suggested a role for mast cell involvement based on the synergistic expression of endothelial and inflammatory genes in response to radiation, including p38a MAP kinase and p65 (NF-κB; Blirando et al., 2011). Given the findings that serine peptidases, particularly tryptases, secreted by mast cells are able to stimulate both fibroblast chemotaxis and collagen production, the potential significance of mast cells in the pathogenesis of fibrosis definitely warrants additional investigation (Hugh and Pemberton, 2002; Caughey, 2007). Furthermore, radiation induced an increase in expression of α_v_β_3_ integrin (Abdollani et al., 2005). The α_v_β_3_ is highly expressed on endothelial cells and is known to have potent angiogenic activity involving proliferation and migration of endothelial cells mediated through FGF and therefore likely contributes to the vascular changes observed in radiation proctitis (Son et al., 2013). Taken together, these mechanisms may individually or collectively contribute to long term defects in tissue and vascular integrity associated with propagating the injury response mechanisms that promote fibrosis (Sheth et al., 2009).

Current treatment options for radiation proctitis vary greatly in success rates (Leiper and Morris, 2007). While there are few, if any, compelling clinical studies in the treatment of radiation proctitis, the majority of therapies are based on the results of small unblinded studies with mixed results. Animal models that utilize focused radiation to the bowel have been effective tools for screening potential compounds because the inflammatory responses that occur in these models accurately represent the diversity seen in patients (Skwarchuk and Travis, 1998; Kang et al., 2000; Symon et al., 2010). The most common first-line therapies have been adapted from the treatments used in IBD, including the anti-inflammatory compounds 5-ASA and steroids, sucralfate which promotes barrier function and metronidazole to mitigate bacterial colony formation (Symon et al., 2010). Increasingly, endoscopic therapies are being employed to control bleeding which include heat probes, lasers, and most commonly argon plasma coagulation (APC). APC involves the flow ionized argon gas and can target small or larger areas of bleeding without making physical contact to the tissue (Leiper and Morris, 2007). Other studies suggest that long term suppression of high pro-inflammatory cytokine levels while also stimulating with pro-survival growth factors will provide the right balance to prevent long term complications (Okunieff et al., 2005). Linard et al. (2013) recently reported a different therapeutic approach focusing on preventing the progression of fibrosis with autologous mesenchymal stem cells (MSCs). Using an irradiated pig model, they initiated infusion of MSCs following endoscopic identification of early fibrosis. MSCs limited the progression of radiation-induced fibrosis due to a reduction in collagen deposition, a decreased transforming TGFβ response and modification of matrix metalloproteinase/TIMP balance. The novelty of this tactic provided a new arena for developing potential therapeutic strategies that may address these currently irreversible complications.

## FIBROSIS ASSOCIATED WITH RADIATION DERMATITIS

Fibrosis of the skin in the context of cancer treatment occurs as a result of radiation-induced dermatitis. Radiation dermatitis is a common side effect of radiation therapy. It has a reported frequency of 85% and is commonly seen in patients receiving radiotherapy for the treatment of the breast, lung, prostate or head and neck cancers. The fibrosis which often accompanies dermatological changes, results in symptoms similar to contractures that markedly compromise patients’ ability to move freely. Examples include patients treated for breast cancer have a limited range of motion of their arm, or head and neck cancer patients who have hindered head movement. The clinical presentation varies from mild erythema to complete epithelial breakdown manifested by painful ulceration and fibrosis (Ryan, 2012). Injury to the skin occurs immediately following the first dose of radiation, disrupting the self-renewing cells of the epidermis (Ryan, 2012). Subsequent doses prevent this cell population from fully replenishing itself and also elicit an inflammatory response resulting in systemic complications. Later doses further exacerbate the alterations in cellular phenotypes and can cause chronic issues including delayed ulcerations, fibrosis and in the most severe cases, necrosis of the tissue (Bey et al., 2010; Salvo et al., 2010).

After an initial exposure to radiation, there is immediate damage to the keratinocyte cells of the skin, which is accompanied by a simultaneous increase in free-radicals, DNA damage and inflammation (Brown and Rzucidlo, 2011; Ryan, 2012). Like other epithelial tissues, many of these biological changes are apparent in the dermis. The TGFβ signaling pathway is largely implicated in driving the pathogenesis of radiation-induced dermatitis where TGFβ levels are markedly increased in irradiated skin and remain elevated for a long period of time (Martin et al., 2000; Flanders et al., 2003). TGFβ isoforms act as potent chemotactic factors for monocytes, neutrophils, mast cells, and fibroblasts. Furthermore, the Smad family of proteins regulates expression of additional genes responsible for inflammation and fibrosis amplifying and maintaining the cascade of inflammatory signals (Flanders et al., 2002). Smad3 null mice exposed to ionizing radiation exhibited decreased skin damage and reduced fibrosis compared to wild type animals, further supporting a critical role for this signaling pathway in the phenotypes associated with radiation dermatitis (Flanders et al., 2002,^2003^).

There is currently no effective intervention to prevent or favorably modify the course and severity of radiation dermatitis (Wong et al., 2013). Treatment strategies have been based on those associated with thermal burns. Consequently, there is an unmet clinical need to develop specific therapies to target the mechanisms underlying the development and persistence of radiation dermatitis (Lataillade et al., 2007; Bey et al., 2010; Salvo et al., 2010; Chan et al., 2012). Pre-clinical animal models of radiation-induced dermatitis in the published literature are limited however, a few recent studies performed in mouse models have shown promising therapeutic efficacy signals. In one example, treatment with a Toll-like receptor 5 agonist reduced radiation dermatitis associated epidermal hyperplasia and dermal inflammation through activation of endogenous antioxidants reducing free-radicals (Burdelya et al., 2011). In another study, the antiallergic agent Azelastine was administered in the food of mice that received an acute dose of radiation and reduced the severity of radiation dermatitis. In these animals there was a modest reduction in the severity of dermatitis. The authors proposed that the mechanism responsible was increased epithelial cell stabilization post-radiation exposure due to preventing the influx of Ca^2^^+^ into the cell resulting in a decrease in free radical generation (Murakami et al., 1997). Until an effective therapeutic is approved for this indication, patients will continue to be treated with standard burn agents such as Aloe Vera which neither prevents fibrosis nor addresses the palliative component associated with skin injury due to radiation exposure.

The clinical observation of heterogeneity in the manifestation of fibrosis-related to radiation-induced dermatitis suggests a possible genetic basis for risk. The results of a recent study by Andreassen et al. (2013) in which cultured fibroblasts from head and neck cancer patients with subcutaneous fibrosis were irradiated. Analysis of the cells provided validation of a group of overexpressed genes associated with a positive fibrosis phenotype. Additional studies on the predictive ability of synergistically acting genes, both in tissue and in peripheral blood, would certainly be of great interest.

## PULMONARY FIBROSIS

Radiation therapy is a critical component in the treatment of many forms of cancers of the lung, breast, and lymphomas. In these cases, the radiated fields include the thorax which exposes the lungs to the potential for injury. Radiation directed to the thoracic region exceeding 50Gy damages pulmonary tissues and may lead to the development of dose-limiting pneumonitis or fibrosis (Reviewed in Tsoutsou and Koukourakis, 2006). Moderate to severe radiation pneumonitis occurs with a reported incidence of between 10 and 20% (Mehta, 2005) and is associated with alveolar damage that causes a significant inflammatory response in the interstitial space of the lung. The increased vascular permeability causes edema and accumulation of proteins in the alveolar space (Rosai, 1996). Pneumonitis develops within 12 weeks of receiving radiation therapy and symptomatically manifests by shortness of breath. Severe cases increase the risk of treatment-related death significantly. Ultimately, pneumonitis may transition to pulmonary fibrosis.

Pulmonary fibrosis is among the most thoroughly studied cancer regimen-induced forms of fibrosis. Although it can be caused by a variety of chemotherapeutic drugs or by radiation and is associated with poor clinical outcomes, its pathogenesis is incompletely understood. Of chemotherapies associated with pulmonary fibrosis, cases resulting from bleomycin treatment are the best studied. Radiation-induced fibrosis has been investigated in parallel and emerging data suggests the biological pathways which impact end organ damage are likely to be similar. Radiation-induced pulmonary fibrosis is mediated through inflammatory cells that are recruited and accumulate in the interstitial space. It is believed that these cells are responsible for the majority of TGFβ production which drives the differentiation of fibroblasts to myofibroblasts depositing collagens into the ECM (Eckes et al., 2000; Wang et al., 2011). In addition, the pulmonary alveolar type II epithelial cells can undergo EMT and differentiate directly into matrix depositing myofibroblasts (Radisky, 2005; Andersson-Sjöland et al., 2011). The process of EMT is mediated by Snail and Twist transcription factors which repress E-Cadherin and promote a mesenchymal phenotype enabling the epithelial cells to escape attachment from the basement membrane permitting anchorage independent growth and migration into the interstitial space (Willis et al., 2005); ultimately, the damaged alveoli collapse and are fully encompassed by connective tissue (Almeida et al., 2013). The process of fibrosis occurs several months later than pneumonitis, typically appearing 6 months after radiation exposure. The timing of these late effects are supported by data showing that increased TGFβ signaling is sustained after pneumonitis resolves thus promoting the latent fibrosis (Rube et al., 2000). Similar signaling events have been described for bleomycin-induced pulmonary fibrosis. The common clinical practice of administering concomitant chemoradiation (in this case, chemotherapy is administered as a radiosensitizer) increases the risk of severe and extended pulmonary fibrosis. Mouse models of thoracic radiation and treatment with bleomycin have revealed molecular mechanisms driving the general induction process of fibrosis in the lung. In addition to the previously described classical TGFβ and TNF-α signaling pathways that are responsible for initiating inflammation at the site of injury, there is subsequent involvement of chemokines and their receptors in orchestrating the recruitment and trafficking of the immune cells from the circulation to the sites of inflammation driving the epithelial cell responses within the alveoli (Johnston et al., 2002; Han et al., 2011). Interestingly, chemokine production differs significantly between the pneumonitic and fibrotic phases of injury suggesting differential roles for subsets of chemokine family members and likely specificity in the immune cells they recruit (Johnston et al., 1998). Whereas chronic inflammation is a hallmark of fibrosis, sustained chemokine recruitment and trafficking of immune cells may lie at the crux of the cellular events propagating the long term complications associated with the condition.

Like the majority of regimen-related toxicities, the risk of pulmonary fibrosis is not ubiquitous among patients undergoing treatment. In reality, despite demographic and treatment intensity equivalence, only a relatively small fraction of patients go on to develop fibrosis. This clinical observation has led to significant interest to determine those factors that are associated with risk. It now appears that genetic differences among patients, especially those genes that are associated with the pathways leading to fibrosis are fundamental determinants of risk. This hypothesis was elegantly demonstrated by Paun and Haston (2012) who compared radiation-induced pulmonary fibrosis susceptibility across a number of mouse syngeneic mouse strains and then, using a genome-wide association study format, identified specific SNP loci associated with the condition. The complexity of the pathobiology of radiation-induced fibrosis and its impact on clinical risk prediction has been further substantiated by candidate SNP approaches. As noted above, TGFβ1 has been associated with the biological sequence leading to fibrosis. Consequently, TGFβ1 SNPs seemingly made a good target for risk determination. Results of substantive clinical studies have been, however, inconsistent (Barnett et al., 2012). It seems likely that effective genomic risk prediction of fibrosis will rely on the discovery of a cluster of SNPs or genes (Petrovski et al., 2009).

Preventive strategies such as the use of intensity-modulated radiation therapy (IMRT) have been suggested to reduce the frequency and severity of radiation induced pulmonary fibrosis (Lavrenkov et al., 2007). On the other hand, modifying treatment-related strategies associated with chemotherapy is more challenging given the wide range of drugs that are associated with lung injury (Carver et al., 2007). Despite attempts to mitigate cancer therapy induced lung injury, pulmonary fibrosis persists and remains a significant side effect that is refractory to most treatments. Presently a lung transplant is viewed as the treatment of choice (Thabut et al., 2003) however, potential biologically directed targets for intervention are emerging. For example, the Foxm1 transcription factor has been implicated in driving inflammation, EMT and proliferation of fibroblasts in the lung and therefore might be of interest as a potential target for intervention (Balli et al., 2013). Additional treatment strategies for pulmonary fibrosis are similar to the previously described therapies for fibrosis from other tissue origins including agents which act as free radical scavengers. One such example approved by the FDA for use as a broad spectrum radioprotective agent is the thiol amifostine (Kouvaris et al., 2007). Clinical trial data suggests that amifostine protects against the long term effects of radiation and chemoradiation induced toxicity to lung and soft tissue including fibrosis. Treatment with amifostine also allows for higher doses of radiation to be administered in situations where that is required for higher tumor kill (Koukourakis et al., 2013). Superoxide dismutases (SODs) are naturally occurring enzymes in the body which act in the early stages of injury. SODs dismutate superoxides to form hydrogen peroxide (H_2_O_2_)and catalase further decomposes H_2_O_2_ to oxygen and water. Because radiation therapy reduces the endogenous SOD, SOD mimetics have been developed for therapeutic use and have demonstrated success in reducing fibrosis by enhancing the activity of antioxidant enzymes in the tissue, preventing oxidative damage with fewer side effects and higher potency than amifostine (Gao et al., 2012; Pan et al., 2012).

## CONCLUDING STATEMENTS

Chronic problems associated with fibrosis are of high importance because they can ultimately force physicians to limit an individuals’ cancer treatment due to a decreased tolerance of the therapies employed. As a result, there is a high demand to develop interventions designed to prevent or treat fibrosis associated with regimen-related toxicities. Animal models designed to mimic radiation and chemotherapy-induced fibrosis in patients and to help understand the exact mechanisms of these pathways have revealed that there are marked differences in the cytokine responses among animal strains. This is consistent with a varied cytokine response seen in patients; therefore highly valuable information has been gained from collecting and incorporating data from several different models. The discovery that many toxicities seem to cluster suggests shared pathoetiologies that will not only assist in the development of such therapeutics, but will also likely result in the use of one therapy for the treatment or prevention of fibrosis in multiple tissue types.

## Conflict of Interest Statement

The authors declare that the research was conducted in the absence of any commercial or financial relationships that could be construed as a potential conflict of interest.

## ABBREVIATIONS

TGFβ: transforming growth factor β
QoL: quality of life
RT: radiation therapy
ROS: reactive oxygen species
SOD: superoxide dismutase
CTGF: connective tissue growth factor
TNF-α: tumor necrosis factor alpha
INF-γ: interferon gamma
EMT: epithelial to mesenchymal transition
Gy Gray: a unit of absorbed dose of ionizing radiation.
